# Humoral and cellular immune responses to Lassa fever virus in Lassa fever survivors and their exposed contacts in Southern Nigeria

**DOI:** 10.1038/s41598-022-26045-w

**Published:** 2022-12-25

**Authors:** Chinedu Ugwu, Testimony Olumade, Ebenezer Nwakpakpa, Venatius Onyia, Elizabeth Odeh, Rosemary Ogonna Duruiheoma, Chiedozie K. Ojide, Matthew Afam Eke, Ifeanyi Emmanuel Nwafor, Nneka Chika-Igwenyi, Augustine M. Abu, Benedict Azuogu, Nnennaya Ajayi, Emeka Ogah, Oluwafemi Ayodeji, Chukwuyem Abejegah, Nelson Adedosu, Nicholas Oyejide, Sylvester Abah, Abiola Omidele, Winifred Ingbian, Emmanuel Osoba, Philomena Eromon, Paul Oluniyi, Olusola Ogunsanya, Anise Happi, Patricia Otuh, Angalee Nadesalingam, George Carnell, Nina Krause, Ernest Aguinam, Rebecca Kinsley, Daniel Matthew L. Storisteanu, Paul Tonks, Diana Nelson, Carley McAlister, Matthew Boisen, Robert Garry, Edward Wright, Nigel Temperton, Simon Frost, Jonathan Luke Heeney, Christian Happi

**Affiliations:** 1 The Africa Centre of Excellence for Genomics of Infectious Diseases (ACEGID), Redeemer’s University Nigeria, Ede, Nigeria; 2 Department of Biological Sciences, Faculty of Natural Sciences, Redeemer’s University, Ede, Osun State Nigeria; 3 Alex Ekwueme Federal University Teaching Hospital (AEFUTHA), Abakaliki, Nigeria; 4 Federal Medical Centre Owo, Owo, Nigeria; 5 Michael Okpara University of Agriculture, Umudike, Nigeria; 6 Lab of Viral Zoonotics, Department of Veterinary Medicine, University of Cambridge, Cambridge, CB3 0ES UK; 7 Zalgen Labs, LCC, Frederick, MD USA; 8 Department of Microbiology and Immunology, School of Medicine, Tulane University, 1430 Tulane Avenue, New Orleans, LA JBJ56870118 USA; 9 School of Life Sciences, University of Sussex, Sussex House, Falmer, Brighton, BN1 9RH UK; 10 Medway School of Pharmacy, The Universities of Greenwich and Kent Medway, Anson Building, Central Avenue, Chatham Maritime, Chatham, Kent, ME4 4TB UK; 11 London School of Hygiene & Tropical Medicine, Keppel St, London, WC1E 7HT UK

**Keywords:** Immunology, Microbiology

## Abstract

Elucidating the adaptive immune characteristics of natural protection to Lassa fever (LF) is vital in designing and selecting optimal vaccine candidates. With rejuvenated interest in LF and a call for accelerated research on the Lassa virus (LASV) vaccine, there is a need to define the correlates of natural protective immune responses to LF. Here, we describe cellular and antibody immune responses present in survivors of LF (N = 370) and their exposed contacts (N = 170) in a LASV endemic region in Nigeria. Interestingly, our data showed comparable T cell and binding antibody responses from both survivors and their contacts, while neutralizing antibody responses were primarily seen in the LF survivors and not their contacts. Neutralizing antibody responses were found to be cross-reactive against all five lineages of LASV with a strong bias to Lineage II, the prevalent strain in southern Nigeria. We demonstrated that both T cell and antibody responses were not detectable in peripheral blood after a decade in LF survivors. Notably LF survivors maintained high levels of detectable binding antibody response for six months while their contacts did not. Lastly, as potential vaccine targets, we identified the regions of the LASV Glycoprotein (GP) and Nucleoprotein (NP) that induced the broadest peptide-specific T cell responses. Taken together this data informs immunological readouts and potential benchmarks for clinical trials evaluating LASV vaccine candidates.

## Introduction

Lassa fever (LF) is an important endemic zoonotic viral hemorrhagic fever disease in West Africa. The etiological agent is a genetically diverse old-world arenavirus called Lassa virus (LASV). There are seven described lineages (I-VII) of the virus circulating across West Africa^1^. Annually, it is estimated to affect about 100,000 people with a case fatality rate reported to be approximately 26% in Nigeria^2,3^. An unprecedented spike in the incidence of LF in Nigeria in 2018 raised global concern^4^. Subsequently, the World Health Organization (WHO) declared LASV an important threat to global health and security requiring urgent countermeasures^5^. The Coalition for Epidemic Preparedness Innovations (CEPI) responded with an accelerated vaccine program. Currently, there are at least two LF vaccines in phase I trials in West Africa with a progressive epidemiological program towards establishing sites for phase III LASV vaccine efficacy trials in five West African countries including Nigeria^6^. Despite the rejuvenated interest in LF and the drive towards developing an effective LF vaccine, the immunology of LF and the correlates of protective immunity to LF are not fully understood. Previous research has shown that strong T cell responses correlated with protection from severe disease, while low or absent neutralizing antibody titres did not correlate with protective immunity^7^. We and others have also demonstrated cross-protective cellular and humoral responses in LF survivors in Nigeria and Sierra Leone^8,9^. While well-defined immunological data are available from animal model studies of LF, there are few human studies. A major limitation is the complex transportation logistics of shipping samples to external labs outside of Africa, often impacting their biological integrity. Through a Biotechnology and Biological Science Research Council (BBSRC)-funded project One health and accelerating Vaccines for Ebola and Lassa fever (project OVEL) project, and a Wellcome Trust intervention fund, we established in-country capacity for immune analysis at the African Center of Excellence for Genomics of Infectious Diseases (ACEGID), Redeemer’s University, Ede, Nigeria. A major challenge to creating a successful LF vaccine is the high genetic diversity of the LASV^10^. Southern Nigeria presents a unique opportunity to evaluate cross-protective immunity to the LASV because of the circulation of the different lineages of LASV endemic in this region. In 2018, we identified genetically distinct (IIa and IIb) sub-lineages maintained in the rodent reservoirs species separated by a major river^4^. Here, we present the first in-country immune analysis study of LF survivors and their contacts in these regions in Southern Nigeria. We evaluated both humoral and cell-mediated immunity from known LF survivors and their contacts in a LASV endemic region in Nigeria. The data from this study is timely and will also provide important immunological benchmarks for upcoming phase II and III clinical efficacy trials in Nigeria.

## Results

### T cell responses in LF survivors and their exposed contacts

**Figure 1:**
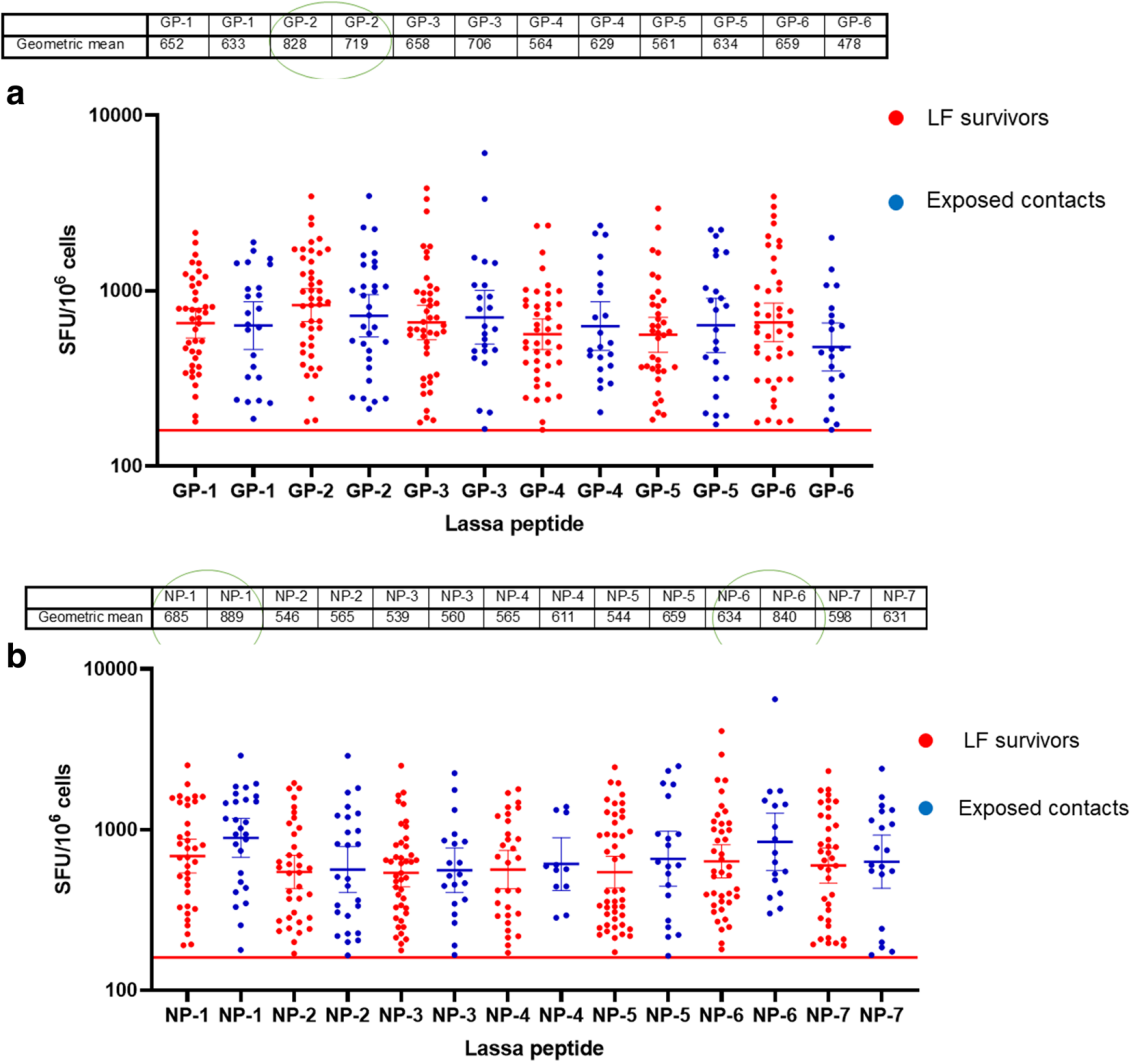
T cell response to LASV GP (**a**) and NP (**b**) proteins from LF survivors and their contacts in Southern Nigeria. PBMCs from either LF survivors (N = 92) or their contacts (N = 50) were stimulated with computationally selected Lassa GP and NP peptides. Interferon gamma ELISpots measured in spot forming units (SFU) per 1.0 × 10^6^ PBMC was used as a read out for T cell response. The result (table showing geometric mean at 95% CI) showed similar T cell response to both GP (**a**) and NP (**b**) peptides from both LF survivors and their contacts. The highest frequency of T cell response was seen in the GP 2 region and NP 1 and 6 regions (green circle). The average limit of detection (red line = 160) was calculated as the mean multiplied by three standard deviations of the three known negative samples.

T cell responses among LF survivors (known LF convalescent patients without LF symptoms or a positive LASV polymerase chain reaction [PCR] test at the time of sample collection) and their contacts (exposed to LASV but without a history of LF symptoms or a positive LASV polymerase chain reaction [PCR] test at the time of sample collection- these are people who may have managed or provided care to LF acute or convalescent patients such as family members, clinicians, nurses etc.) were evaluated using direct ex vivo interferon gamma (IFNγ) T cell ELISpot. LASV GP and NP 15 mer peptides from consensus sequences reflecting common LASV lineages were generated using data from our LASV whole genome sequencing from the study sites in Nigeria and combined with other international genomic databases^4,11^. The GP peptides were divided into 6 pools and the NP into 7 pools. Peptides were incubated with PBMCs from either survivors of LF or their contacts in culture overnight and IFNγ spots were counted as a read-out for active T cell response (spot forming unit per 1 million cells). The average limit of detection (red line = 160) was calculated as the mean multiplied by three standard deviations of the three known negative/naive samples (individuals with no known previous exposure to LASV)^12^. Of the 142 samples (92 survivors and 50 contacts) analyzed for their T cell responses, 65% survivors and 66% of their contacts were found to have T cell responses to either GP and NP, or both peptides pools above detectable threshold for confirmed naive/negative individuals. Thus, the result showed similar magnitude of T cell response to both GP and NP proteins in both survivors and their contacts. The highest frequency of T cell response was seen in the NP pool 1 (NP 1-91) and 6 (NP 411–491) amino acid regions and the GP 2 (GP 92–172) amino acid region (Fig. 1a,b) from both survivors and their contacts.

### LASV neutralizing antibodies responses were predominantly in LF survivors, while LASV binding antibodies was found in both LF survivors and their exposed contacts

**Figure 2:**
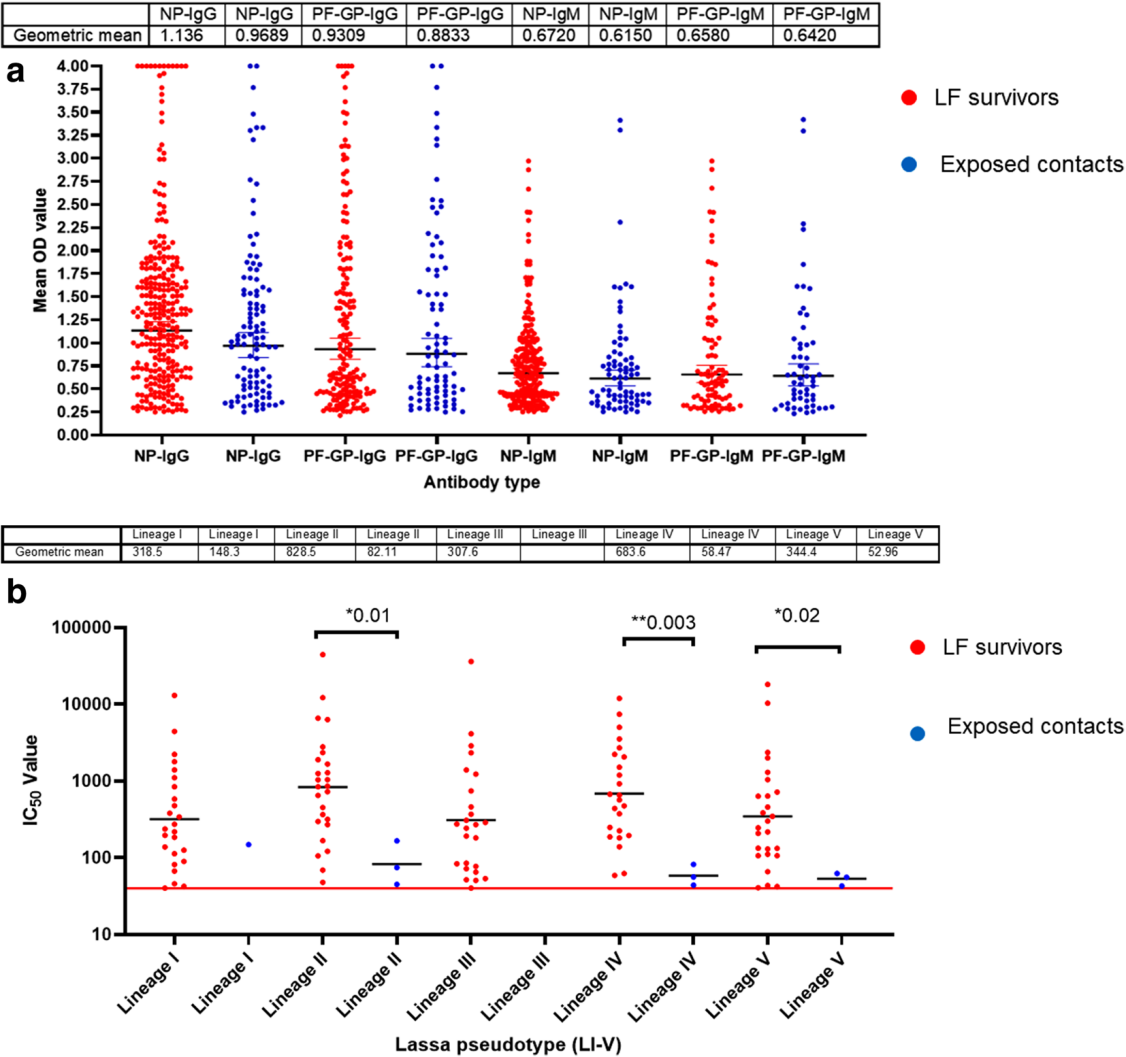
Binding antibody responses (IgG and IgM) to LASV GP and NP proteins (**a**) and neutralizing antibody response to LASV PVs (Lineages I-V) (**b**) from LF survivors and their contacts in Southern Nigeria. Specific Lassa GP and NP ELISA was used to measure the binding antibody response (IgG and IgM) from LF survivors (N = 370) and their contacts (N = 170). We used a 1:100 dilution of the serum sample. Among those with a binding antibody response, we selected some sera from LF survivors and their contacts and measured the neutralizing titre (IC_50_) against LASV PV expressing the LASV GPC of different lineages ((Lineages I–V). The OD of the negative cutoff was selected as the mean multiplied by three standard deviations of three known negative samples (0.25) for binding antibody and 40 for neutralizing antibody response (the limit of detection). The table shows geometric mean at 95% CI. Statistical significance was calculated by Mann–Whitney test and p values are indicated. (Capped line with * indicating significance).

Using the standard protocols and validated kits (ReLASV Pan-Lassa NP IgG/IgM ELISA kit and the prototype ReLASV Pan-Lassa prefusion GP IgG/IgM ELISA Kit (Zalgen Labs, LLC)), we estimated the binding antibody responses (IgG and IgM) of LF survivors and their contacts^13,14^. Of the 540 sera tested (370 survivors and 170 contacts) both survivors and their contacts had similar percentages of both IgG and IgM responders to both LASV GP and NP proteins (Table 1). The mean OD was not significantly different between the survivors and their contacts (Fig. 2a). We then randomly selected 89 samples (59 survivors and 30 contacts) from those with binding antibodies and measured their neutralizing potential against 5 different LASV pseudotype viruses (PV) expressing the GPC of Lineages I–V. Sera from the randomly selected sub-cohort were measured for evidence of neutralizing antibodies against the LASV PV panels. Notably, while 40% of the survivors had neutralizing antibodies against one or more of the LASV PVs, only 6% of their contacts had detectable neutralizing antibody responses. This was reflected in the mean IC_50_ detected, which was significantly higher for the LF survivors compared to their contacts across the five LASV lineages (Fig. 2b). Importantly, none of the contacts had neutralizing antibodies to LASV pseudotype Lineage III, another major circulating LASV strain in Nigeria (Fig. 2b).

**Table 1:** The seroprevalence of LASV GP and NP binding antibody (IgG and IgM) response among survivors and their contacts in Southern Nigeria.

Sub-population	NP-IgG (%)	PF-GP-IgG (%)	NP-IgM (%)	PF-GP-IgM (%)
LF survivors	77	48	32	23
Contacts	64	50	48	33

### LF survivors retain circulating binding antibody responses for approximately six months

**Figure 3:**
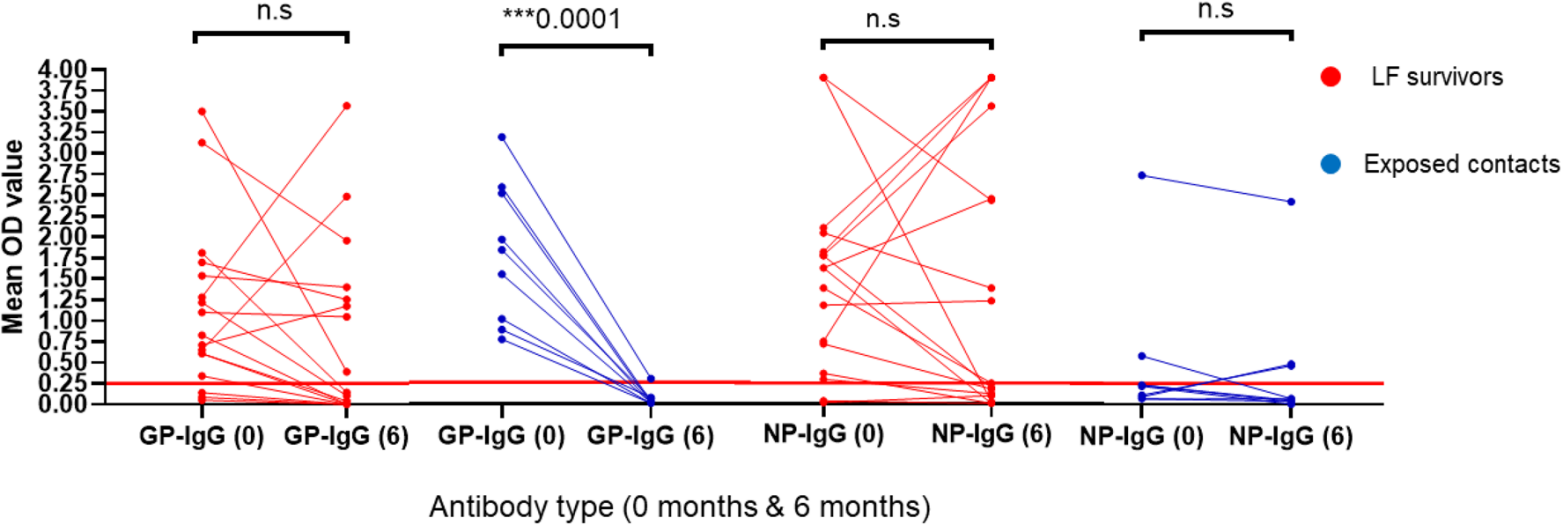
Binding antibody responses (IgG) to LASV GP and NP proteins from LF survivors and their contacts in Southern Nigeria six months post-infection. Durability of the binding antibody response (IgG) of a few LF survivors (N = 17) and their contacts (N = 9) was measured six months after the first sample collection using same specific LASV GP and NP ELISA (1:100 dilution). While most of the survivors retain their binding antibody responses, their contacts didn’t after six months. All the contacts lost their anti-GP IgG and only one retained anti-NP IgG. Statistical significance was calculated by Mann–Whitney test and p values are indicated. (Capped line with * indicating significance). The OD of the negative cutoff was selected as mean multiplied by three standard deviations of three known negative samples (0.25) for binding antibody (red line = 0.25).

Our effort to longitudinally follow up our participants to measure the durability of both cellular and humoral immune responses every six months for a period of two years was hampered by several challenges such as a difficulty in tracking participants, the social stigmatization of LF survivors and restrictions in place during the COVID19 pandemic. However, we were able to follow a limited cohort of 17 LF survivors and 9 contacts six months after their first sample collection. While the majority of the LF survivors retained their IgG antibody responses to both the GP and NP after six months, we did not find this in the LF-contact cases studied (Fig. 3). None of the LF-contacts had detectable levels of anti GP IgG (P = 0.001) while only one LF-contacts had anti NP IgG after six months (1/9) following LF exposure.

### T cell and Antibody responses to LF wane in less than a decade

**Figure 4:**
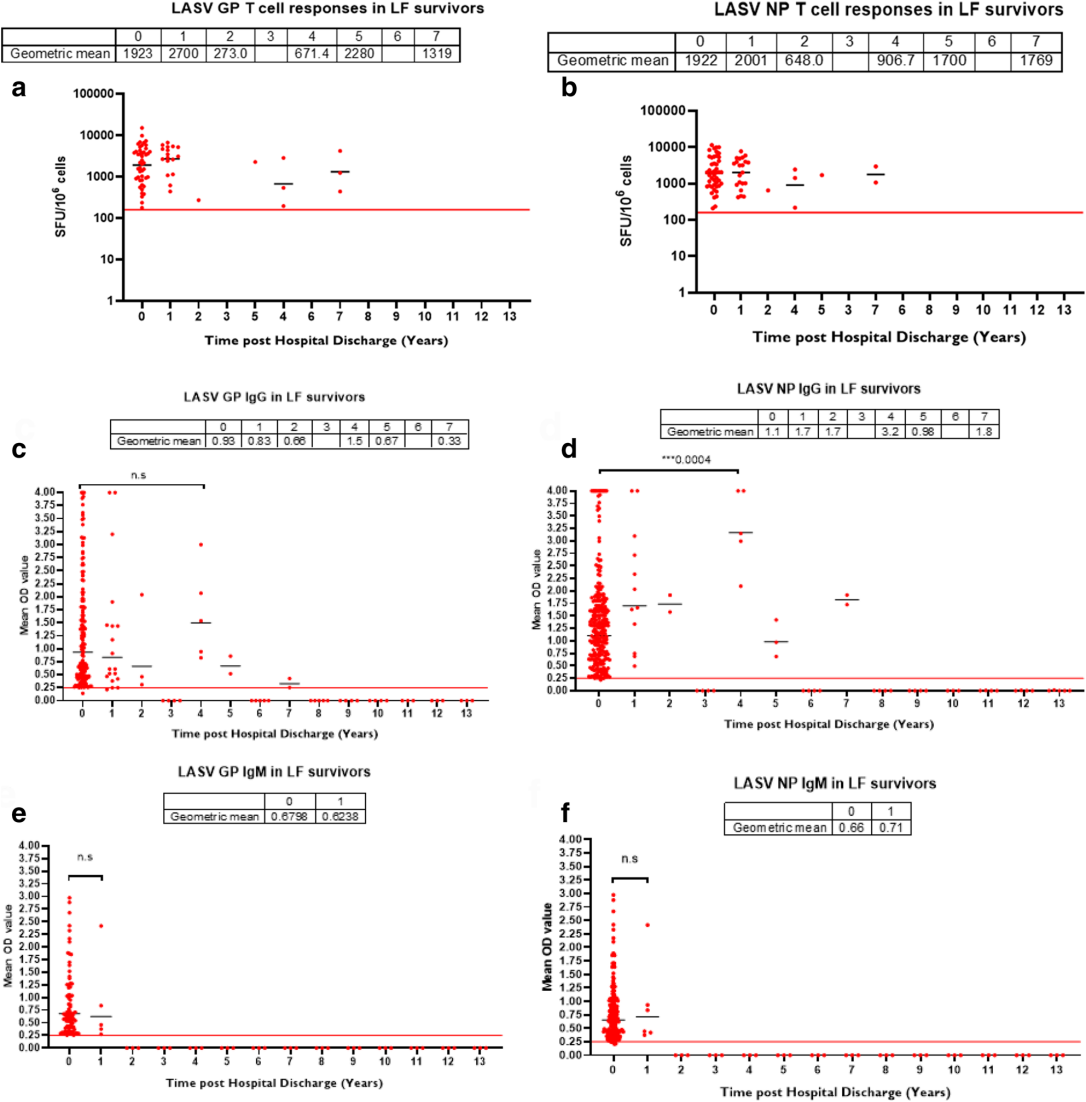
T cell and antibody responses to LASV wane after a decade in LF survivors. Durability of both T cell response (positive specific interferon gamma ELISpot (**a**,**b**)) and antibody response (positive ELISA (**c**–**f**)) was measured by subtracting the time the patient was discharged from hospital (confirmed LASV negative RT-PCR test) from the time of sample collection (time in years) over 13 years. There was no T cell response after 8 years post-hospital discharge and IgG to both LASV GP and NP waned after eight years while IgM waned after 1 year post-infection. The table shows geometric mean at 95% CI. Statistical significance was calculated by Mann–Whitney test and p values are indicated. (Capped line with * indicating significance).

To address the limitations associated with the targeted 6 monthly longitudinal follow-up, we studied the durability of immune responses by examining the levels at the time of hospital discharge (i.e. the first negative RT-PCR test) and the time of sample collection (in years). Analysis revealed that the majority of T cell responses were detected in recent survivors (0–1-years after discharge from hospital) while T cell responses were not detected in peripheral blood in individuals tested 8 years after hospital discharge (Fig. 4a,b). Similar to the T cell response data, there was no detectable serological antibody response in individuals 8 years post infection. Serum IgG binding responses to both LASV GP and NP waned after eight years while IgM responses, as expected, were absent one year post-infection (Fig. 4c–f) The peak mean IgG binding antibody response was seen amongst individuals tested four years post-infection, which was significant for anti-NP antibodies (P = 0.001) and not for anti-GP antibodies.

## Discussion

Lassa fever is an important viral hemorrhagic fever endemic to West Africa that has been neglected in the decades following its discovery and characterization. After the WHO recognized LASV as a priority pathogen in need of accelerated research for vaccine development, there has been a rejuvenated interest in LF with a need to better understand the immune correlates that distinguish non-progressors (i.e., LF-contacts) from progressors (clinical LF) to facilitate vaccine development^5,15^. Currently, there are several vaccine candidates in early stage clinical trials in West Africa where LASV is endemic. Despite this progress, there is still a paucity of information describing protective immunity to LASV. A deeper understanding of the immune correlates of protection are needed as immune benchmarks for LF vaccines progress from phase 1 to phase 3 trials. Here, we compared antibody and T cell immune responses from LF survivors and their direct contacts exposed in a highly LASV-endemic region in Nigeria. Our data provide evidence of the presence of pre-existing immunity in LASV endemic regions in Nigeria. This was enabled by careful case definition and interviews with contacts (exposed non-convalescent participants) that had no history of clinical symptoms of LF at the time of sampling. Importantly, we determined that they had similar levels of T cell and binding antibody IgG responses (Fig. 1a,b). This is consistent with previous epidemiological studies that have shown that up to 80% of LF cases are asymptomatic^3^. Though, the binding antibody responses are similar in both LF survivors and their contacts, only patients who had recovered from LF were found to have generated detectable levels of neutralizing antibody responses (Fig. 2a,b). Moreover, while most of the LF survivors retained their serum binding antibodies after six months, their contacts lost detectable antibodies by 6 months (Fig. 3) post-exposure. The absence of detectable neutralizing antibodies and loss of binding antibody responses after six months amongst the LF-exposed contacts may be due to the high levels of LASV viremia in LF patients as compared to abortive or low-level infection in LF-exposed individuals, similar to what has been reported for SARS-CoV-2 infection^16^. These findings warrant further immuno-epidemiological studies to determine if prior exposure to LASV is protective in individuals with neutralizing antibody responses, or if T-cells and low-level binding antibody responses are sufficient to protect from clinical disease severity.

In LF survivors, we observed that T cell and antibody (binding and neutralizing) responses were present at an early-stage after hospital discharge. T cell responses were present 1–2 years after infection, and antibody responses remain for at least 4 years (Fig. 4a–f). Although this study was not designed as a detailed follow-up study, this is the first description of immune responses to LASV in survivors spanning over ten years. These findings provide important new insights into the durability of both the humoral and cellular immune responses to LASV over a decade. The lack of both T cells and antibody responses to LASV eight years post-infection suggests that vaccine boosting may be needed for high-risk populations to sustain a protective level of immune responses over time, should vaccination produce a similar immune response to infection. Furthermore, considerations of vaccine trial endpoints, such as protection from infection versus protection from disease will be important considerations and to determine if T-cell immunity is the key protective mechanism of protection from clinical LF, but not community transmission. Other vaccine trial endpoint considerations should include the burden of post-infection complications such as sensorineural loss which is seen in both asymptomatic and symptomatic LF patients^17^.

In summary, our data confirm that LF survivors generate detectable neutralizing antibody responses, in contrast to previous studies where they were found later post infection^7^. Similar to our previous studies, our data also confirmed cross-reactive antibody responses to different LASV lineages (I-V) in sera from LF survivors and their contacts in Southern Nigeria^8^. The LASV Lineage II is the most prevalent lineage of LASV in Southern Nigeria and was found to be the predominantly neutralized lineage by the survivor’s antibodies. It is of interest for future research to see if the two types of Lineage II (a&b) seen in different parts of Southern Nigeria (Southwest and Southeast) induce varying and/or overlapping immune responses. We also confirmed that certain regions of the LASV NP 1 and 6 and GP 2 generated the broadest T cell responses among our cohort of LF survivors and their contacts in Southern Nigeria. The sequences of NP 1 and 6 regions recognised are similar to the conserved region described by Sullivan et al. in Nigerian LF survivors^9^. Other conserved regions of the nucleoprotein with similarity to previously reported sequences of the LASV NP were NP2,3,7 (NNQFGTMPSLTLACL, QTMDGILKSILKVKR, LCHMHTGVVVEKKKR)^18^. Further research to identify the optimal conserved region/epitopes as vaccine targets will be valuable for vaccine efficacy against diverse lineages, given the major role that T cell responses appear to play as a major correlate of protection for LF disease.

In summary, this study is the first description of both naturally acquired humoral and cellular immune responses to LASV in Nigeria. This in-country immune-correlate study has helped us build local capacity for immunological assays for upcoming vaccine clinical trials for LASV disease prevention, as well as other infectious pathogens such as SARS-CoV-2. As early phase LASV trials expand and progress into high-risk populations, the role of natural immunity versus vaccine-induced immunity to LF will be important to delineate.

### Limitation of the current study

Due to the COVID19 pandemic, we were only able to follow a subset of our participants over the full period of the study.

## Methods

### Study participants

The study participants were enrolled through ACEGID Clinical site networks in Abakaliki (the Alex Ekwueme Federal University Teaching Hospital (AE-FUTHA) Abakaliki Ebonyi State) and Owo (Federal Medical Centre (FMC) Owo, Ondo State) between October 2018 and October 2020. These are two major LASV endemic regions in Southern Nigeria (We were unable to recruit participants from Edo State another major LASV endemic region in Southern Nigeria, because of a lack of security and a delay in ethical approval from the hospital). The study comprised two categories of participants: known LF convalescent patients (survivors N = 370) with a negative LASV polymerase chain reaction [PCR] tests at the time of sample collection and their close contacts (exposed asymptomatic participants N = 170).

There were more males (54.5%, 57.1%) than females (45.5%, 42.9%) in both LF survivors and their contacts and the mean age for both survivors and their contacts were 32.59(± 14.552) and 38.14(± 13.329) at 95% confident interval respectively.

### LASV glycoprotein (GP) and nucleoprotein (NP) consensus peptides

Different LASV lineages sequences obtained from our previous whole genome studies and internationally shared genomic databases (Josiah NP (NCBI accession number NP_694869) and GPC (NCBI accession number NP_694870) and strain GA391 GPC (NCBI accession number CAA36645)) were aligned using a multiple sequence alignment program (MAFFT)^4,11,19^. A single consensus sequence with maximum coverage of 11mers for each of the NP and GP was obtained using Epigraph (https://www.hiv.lanl.gov/content/sequence/EPIGRAPH/epigraph.html) from the aligned input option and Bespoke Python script was used to generate the overlapping 11mers (140 NP (pooled into 7 peptide group of 15 mer amino acid), and 120 GPC (pooled into 6 peptide group of 15 mer amino acid)) from the epigraph sequences. The consensus GP and NP sequences were then commercially synthesized for use in ELISpot assay (Mimotopes).

### PBMC isolation and serum separation

Plasma and peripheral blood mononuclear cells (PBMCs) were separated immediately following manufacturer instructions (Sigma‐Aldrich, Z642843). 20 mL of whole blood was transferred from the EDTA tubes into LeucoSep-tube containing ficoll-hypaque at a ratio of 2:1. The tube was centrifuged at 800×g for 30 min at room temperature in a swinging-bucket rotor with no break. The top layer of plasma was removed, and the buffy coat interface was collected, washed twice with PBS-EDTA (10 mM), and centrifuged for 10 min at 250×*g* with the brake on. The pelleted cells were suspended in red blood cell lysis buffer (1 mM KHCO3, 0.15 M NH4Cl, 0.1 mM EDTA, HCl pH 7.2 to 7.4) at room temperature for 5 min. The cells were washed again with PBS-EDTA, centrifuged at 250×*g* for 10 min at 4 °C and resuspended in appropriate medium (Leibovitz medium, Sigma-Aldrich, L1518) for further assay (ELISpot). The plasma was centrifuged at 250×*g* for 5 min at 4 °C and transferred to a new 15 mL tube to remove cells and debris. Both the PBMCs and plasma were transferred to 2 mL cryotubes for further assay (ELISA) and storage at − 80 °C.

### ELISpot

PBMCs were re-suspended in 10 mL of media (Leibovitz media supplemented: 5 mL Pen/Strep, 5 mL l-glutamine, 12.5 mL HEPES, 0.5 mL 2-mercaptoethanol) and were plated onto customized ELISpot plates (Catalogue no: 10602KMM) coated with IFNγ (2 × 10^5^ cells/ well). 50 µL (1 mg/mL) of each of the LASV GP or NP peptides (described previously), anti CD3 and vehicle control (media) was then added separately to individual wells of the customized ELISpot plates containing the PBMC (anti CD3 and vehicle control (media) were used as both positive and negative controls respectively). The plate was incubated in the class-II cabinet for 20–24 h at 37 °C and 5% CO_2_ with no disturbance. After incubation, 80 µL of detection solution was added to each well and incubated for 2 h at room temperature following washing twice with 0.05% Tween-PBS. Thereafter, the detection solution was decanted and wells were washed three times with 0.05% Tween-PBS and incubated with 80 L of tertiary solution for another 30 min at room temperature. The plate was later washed two times with 0.05% Tween-PBS and two times with distilled water, 200 µL/well each time. 80 L/well of blue developer solution was added and incubated for 15 min at room temperature. The reaction was stopped by gently rinsing the membrane with tap water, and decanting; this step was repeated three times. The protective underdrain was removed and the back of the plate was also rinsed with the tap water. The plate was air-dried for 24 h face down on paper towels on the bench top. Scanning and plate count was done using CTL immunospot counter.

### ELISA

ELISA was performed on human plasma using ReLASV® Pan-Lassa IgG/IgM ELISA Test Kit (Zalgen Labs, LLC) with either GP or NP as the capture antigen according to the manufacturer’s instructions. Lyophilized human monoclonal calibrator and negative control plasma were reconstituted with 0.25 mL laboratory-grade water. Calibrator was diluted 1:101 (0.01 mL/1.0 mL followed by four threefold serial dilutions to create a calibration curve for antibody concentration estimation. Calibrator (or Reference) dilutions, diluted negative control and patient samples were transferred (0.1 mL/well) in duplicate wells. Microwell plates were incubated at ambient temperature (18–30 °C) for 30 min. Microwell plates were washed four times with 0.05% Tween-PBS wash buffer. Anti-human IgG or IgM-horse- radish peroxidase conjugated reagent was added to each well (0.1 mL/well) followed by a 30 min incubation at ambient temperature. After repeating the PBS-Tween wash, 3,3′,5,5′-Tetramethylbenzidine (TMB) Substrate was added to each well (0.1 mL/well). The TMB substrate was incubated for 10 min followed by the addition (0.1 mL/well) of Stopping Solution (2% Methane sulfonic Acid). Developed ELISA plates were read at 450 nm (with 650 nm reference). IgG or IgM concentration was estimated using the Optical Density (OD) reading from the ELISA plate reader. The negative cut-off was determined as the mean multiplied by three standard deviations of three known negative samples (mean(3SD) three samples from participants with no prior exposure to LF).

### Virus pseudotype neutralization assay

LASV PV expressing the LASV GPC of different lineages ((lineages I–V) (produced by LVZ University of Cambridge UK) on the surface of a particle containing the core proteins of HIV (Lentivirus based) were used. These are capable of a single round of replication and were used to measure the neutralizing activity of both the LF survivor’s sera and their contact’s sera according to an established protocol. Briefly, twofold serial dilutions of plasma were performed in a Nunc™ F96 MicroWell™ white polystyrene plate and 50 µL of the LASV PV solution with a concentration 1 × 10^6^ relative luminescence units (RLU) were added to each well. The plate was incubated at 37 °C, 5% CO_2_ for an hour. Afterwards 50 µL of 1.5 × 10^4^ HEK293T/17 cells were added to each well and incubated at 37 °C, 5% CO_2_ for 48 h. Firefly luciferase gene expression was evaluated using a commercially available reagent (Promega BrightGlo)^20^. Luminescence was measured using the Promega Explorer Luminometer.

### Data analysis and statistical methods

Data was analyzed using Microsoft Excel (version 16.39, Microsoft, Redmond, WA) and Prism (version 8.4.2, 2020, GraphPad Software, Inc., San Diego, CA). Continuous variables were compared using Mann–Whitney non-parametric tests and the geometric mean was used for descriptive statistics. For all statistical analyses, P value < 0.05 was considered significant at 95% confidence interval (C.I.).

### Ethical approval

All methods were carried out in accordance with relevant guidelines and regulations. All subjects enrolled in this study and/or their legal guardians provided written informed consent. Human subjects testing and sample collection, was approved by the Redeemer’s University Institutional Review Board, the Nigerian National Health Research Ethics Committee, Federal Medical centre (FMC), Owo, Alex Ekwueme Federal Teaching Hospital (AE-FUTHA) Ethics and Scientific Research Committee and the University of Cambridge Institutional Review Board. Once informed consent is obtained from the participants, blood samples were collected from study participants and processed in the Virology Laboratory at the Alex Ekwueme Federal University Teaching Hospital (AE-FUTHA) Abakaliki Ebonyi State and Federal Medical Centre (FMC) Owo, Ondo State. Only qualified Nigerian medical personnel and laboratory staff were involved in the administration of questionnaire and sample collection from the participants.

## Data Availability

The datasets generated during and/or analyzed during the current study are available from the corresponding author on reasonable request.
